# How Did Women From South Asian Backgrounds and People Seeking Asylum and Refugees Look After Their Health and the Health of Others During the COVID‐19 Pandemic? A Community Research Approach

**DOI:** 10.1111/hex.70717

**Published:** 2026-06-21

**Authors:** Olivia Joseph, Nadia Ait Yahya, Lilly Butt, Amana Khan, Sabiya Khan, Jane K. O'Hara, Abigail Albutt, Ryan Carter, Rameen Haq, Gemma Louch

**Affiliations:** ^1^ NIHR Yorkshire and Humber Patient Safety Research Collaboration Bradford Institute for Health Research Bradford UK; ^2^ School of Psychology University of Leeds Leeds UK; ^3^ The Healthcare Improvement Studies Institute (THIS Institute), Department of Public Health and Primary Care University of Cambridge Cambridge UK; ^4^ Institute of Clinical Trials Research (LICTR) University of Leeds Leeds UK; ^5^ School of Healthcare University of Leeds Leeds UK

**Keywords:** community research, COVID‐19, healthcare inequality and inequity, qualitative

## Abstract

**Background:**

The COVID‐19 pandemic had a profound impact on people's lives globally and affected access to, utilisation and the delivery of healthcare. Many communities were disproportionally affected by the pandemic. Community‐based research approaches may help address potential healthcare inequities by identifying and understanding people's experiences and needs relating to healthcare access and utilisation.

**Methods:**

For this qualitative study, members of community organisations were trained and supported to interview women from South Asian backgrounds and people seeking asylum and refuge about their experiences of accessing healthcare services and how they looked after their health and the health of others during the COVID‐19 pandemic. Twenty‐four people took part in a semi‐structured interview from four community organisations in West Yorkshire, UK between July and December 2021. The approach to analysis was inductive, using reflexive thematic analysis.

**Findings:**

Three themes were generated, each with its own subthemes: (1) COVID‐19 magnifying existing challenges; (2) Adjusting to shifts and exclusions in healthcare, and (3) Healthcare experiences and trust. Profound psychological and social impacts were evident, with COVID‐19 compounding existing life challenges. There was a commitment to following protective actions, despite perceptions of unclear and inadequate Government public health messaging. There were examples of extreme environmental challenges and using adaptation and flexibility to navigate the healthcare system. Perceived limitations of healthcare services were often ‘bridged’ through the support and advocacy of family and community members.

**Conclusion:**

Our findings highlight the significant impact of the COVID‐19 pandemic on women from South Asian backgrounds and people seeking asylum and refuge, the inequality and inequity experienced whilst navigating healthcare services and looking after their health and the health of others during the pandemic, and how people adapted to rapidly evolving ways of engaging with the healthcare system. Investment in on‐going community partnerships is essential to identify and work with the strengths, skills, resources and knowledge within communities to respond when needs arise.

**Patient or Public Contribution:**

Community researchers were part of the research team and involved in all phases of the research, including reviewing study documentation, participant recruitment and data collection, interpretation of the data and co‐authoring this paper.

## 1 Background

The COVID‐19 pandemic has had a profound impact on people's lives globally [1, 2], affecting access to healthcare and healthcare utilisation [3, 4, 5] and the delivery of healthcare services [6, 7, 8, 9].

Many communities were disproportionally affected by the COVID‐19 pandemic due to longstanding structural inequities [10]. Empirical evidence shows that racialised and minoritised ethnic groups and people seeking asylum and refuge at varied stages of resettlement, were at risk of health inequalities due to systemic barriers in housing, employment, legal status, access to healthcare and exposure to discriminatory practices [11, 12, 13, 14, 15, 16, 17, 18]. These heterogenous groups often share exposure to structural and social determinants that may systematically increase vulnerability during public health emergencies. Additionally, these determinants can intersect to compound vulnerability and shape differential experiences [15].

In the UK and internationally, research highlighted the disproportionate impact of the COVID‐19 pandemic on socially vulnerable groups. For example, barriers to accessing healthcare such as fear and distrust, reduced provision and remote services for people experiencing homelessness and people facing challenges due to their immigration status, alongside barriers to accessing and following guidance such as digital exclusion and problems with accommodation for these groups [19, 20]. A systematic review of migrants in high‐income countries found that migrant populations were disproportionately represented among COVID‐19 cases and deaths, with undocumented migrants, migrant healthcare workers and those living in camps particularly affected [20]. The review identified risk factors including overcrowded accommodation, high‐risk occupations, language barriers, inadequate information and reduced healthcare entitlement. Research from other international contexts similarly identified barriers during the COVID‐19 pandemic, which exacerbated challenges in accessing healthcare for ethnic minority and migrant women in England, Canada and Australia, including barriers relating to institutions, community perceptions and socio‐economic factors [14, 21, 22], and evidence suggests that migrant groups, who may have already faced barriers to accessing primary care, experienced digital exclusion as a result of the COVID‐19 pandemic [23].

Democratising research involves the intentional redistribution of power ensuring that diverse communities shape research priorities, processes and outcomes, this was recognised as crucial, and particularly relevant during the early stages of the COVID‐19 pandemic, when exclusion from decision‐making exacerbated inequities [24, 25]. Relatedly, the potential benefits of community‐based research approaches that aim to partner with community members to identify and address community needs [26] are recognised, especially in times of crisis [27] and in the context of health disparities and health equity [28]. Indeed, a report on ethnic health inequalities and the NHS published by the King's Fund [29] commissioned by the NHS Race and Health Observatory, recommended increased focus and investment in community engagement to understand and address ethnic health inequalities. The report emphasised the importance of developing sustained and trusted relationships with communities, putting community voices at the heart of how services work and building and mobilising community assets. Furthermore, the 2020 – 2021 World Health Organisations COVID‐19 Global Risk Communication and Community Engagement Strategy emphasises the need for community‐centred, participatory approaches to alleviate stress and fear caused by the uncertainty of the pandemic [30].

We present findings from a study that adopted a community research approach to understand how women from South Asian backgrounds and people seeking asylum and refuge (at different stages of resettlement) accessed and engaged with health services and how they maintained their own health and the health of others during the COVID‐19 pandemic. These groups were selected because they were the primary service users of the community organisation partners in this study and because of the well‐documented structural barriers and disproportionate harm experienced by these groups in relation to healthcare and health outcomes [14, 15]. The study aimed to generate evidence‐informed recommendations for policy makers, healthcare systems and the healthcare workforce, to develop more equitable, culturally responsive, and safety‐enhancing future pandemic responses.

## 2 Methodology

### 2.1 Community Research Approach

We adopted a community research approach grounded in participatory and action research traditions, which promotes shared decision‐making and meaningful involvement of community members throughout the research [31, 32]. In our research, we use the term ‘community researcher’ to reflect the members of the research team who were from organisations that related to, represented and engaged with the communities of interest.

We chose this approach because researchers who are external to these communities may lack the relational knowledge, cultural understanding and contextual awareness required to interpret participants’ experiences accurately and sensitively. These limitations can include poor insight into cultural norms, migration histories, community dynamics and potential power imbalances, which may inhibit open dialogue. Community researchers bring insider insight and established relationships that enhance the relevance and equity of the research. It is vital, where possible, to ensure participants are in a comfortable, safe environment to develop rapport, contributing to rich and authentic data [33, 34]. The idea for this research was developed in the early stages of the COVID‐19 pandemic. The team were able to respond quickly by harnessing existing connections and collaborations with local community organisations to explore whether organisations would be interested in the research topic, and in supporting the development of a community research approach.

### 2.2 Community Researchers: Collaboration, Training and Support

We partnered with community organisations based in West Yorkshire, UK to support recruitment and interviews, and the participant groups reflected the primary service users of these organisations. This included:

1. Women from South Asian backgrounds (Organisation 1)
2. People seeking asylum and refugees (Organisations 2, 3 and 4)

Up to two people per organisation, affiliated with the four organisations, participated in two half day interactive training sessions (1 week apart with activities within the intervening period) commissioned by the team, and delivered by an experienced qualitative researcher. The training covered:

- What is qualitative research?
- Fundamental assumptions of qualitative research
- Basic interviewing technique
- Managing different interview situations

The training aimed to provide the community researchers with the tools, practical support and skills to conduct interviews with the communities of interest. Following the training, four community researchers representing four organisations went on to conduct interviews. Each community researcher was paired with an experienced researcher, to provide individual support on an ongoing basis. This included regular check‐ins and debriefs after recruiting and interviewing participants, as well as practical guidance and documentation, including signposting to relevant mental health and support services, safeguarding information and procedures to follow if a participant indicates they are feeling distressed or showed signs of distress during the research. The community researchers were trained and supported to obtain informed consent from interview participants. To ensure consistency in the support provided, practical guidance and documentation was produced for the paired researchers supporting community researchers.

### 2.3 The Research Team

The team comprised four female community researchers from organisations that related to, represented and engaged with the communities of interest (NA, LB, AK, SK) and six researchers (five female, one male) affiliated with one healthcare research organisation in West Yorkshire, UK (GL, OJ, AA, RH, RC, JOH). The researchers affiliated with the healthcare research organisation had a range of research experience including Health Services Research, Psychology, Patient Safety, Patient and Public Involvement and Engagement and included two research assistants, three research fellows, one senior research fellow and one senior academic.

### 2.4 Design

Critical theory served as the theoretical foundation for this research [35], providing a lens through which to examine issues of equity, social justice and the ways healthcare structures and power relations shape access to health services during the COVID‐19 pandemic [36]. Guided by this paradigm, we adopted a qualitative approach using semi‐structured interviews to explore how participants navigated and experienced healthcare. This approach allowed us to attend simultaneously to individual, micro level experiences and the wider macro level influences of health systems and societal structures. The topic guide covered participants’ general health and wellbeing; their use of healthcare services before and during the pandemic; key timepoints in relation to COVID‐19 and health behaviours; and experiences of COVID‐19 public health guidance (e.g. government and NHS communications). Community researchers contributed to the development and phrasing of the topic guide to ensure the cultural and contextual appropriateness of its questions. Our approach was underpinned by the concept of information power and the items and dimensions conceptualised by Malterud et al [37].

### 2.5 Setting and Participant Information

Twenty four participants were recruited between July and December 2021.

All participants took part in one interview. High‐level descriptive characteristics were provided by 18 participants, for six participants demographic information was not provided, or was partially provided (see Table 1). To ensure participant anonymity, we have not reported individual participant demographic information. Participants were asked to describe their ethnicity in their own words.

**Table 1:** Participant demographic information.

Age	*Provided by n* = *19*
	18‐24 (n = 6)
	25‐34 (n = 2)
	35‐44 (n = 6)
	45‐54 (n = 3)
	55‐64 (n = 1)
	65 or over (n = 1)
Gender	*Provided by n* = *24*
	Female (n = 18)
	Male (n = 6)
Ethnicity/Nationality	*Provided by n* = *18*
	African (n = 2)
	Arab (n = 1)
	Asian (n = 1)
	Asian Pakistani (n = 1)
	British Pakistani (n = 5)
	Honduran (n = 1)
	Kurdish (n = 1)
	Latin American (n = 1)
	Middle Eastern (n = 1)
	Middle Eastern Asian (n = 1)
	Rohingya (n = 1)
	Spanish American (n = 1)
	Syrian (n = 1)
Women from South Asian backgrounds	n = 9 (organisation 1)
People seeking asylum and refugees	n = 15 (organisations 2, 3 and 4)

### 2.6 Procedure

Community researchers invited potential participants through their existing relationships, organisational contacts, and wider community networks, via convenience‑sampling. Eligibility criteria were broad and included anyone aged 18 or above. Potential participants received a written information sheet and verbal explanation of what the research involved. Community researchers drew on their understanding of their service users’ circumstances, together with the informed‑consent training and guidance provided, to determine whether individuals were able to understand the study and felt comfortable participating. Verbal consent was obtained and recorded separately to interview data. Semi‐structured interviews were conducted by the four community researchers (NA, LB, AK, SK) at times mutually convenient to researchers and participants. These were conducted face‐to‐face or virtually (using Zoom^1^ online meeting software‐ audio function only) according to researcher and participant preference. This approach adhered to COVID‐19 guidance at the time. Participants received a £20 shopping voucher for their time. The 24 interviews lasted between 22 and 69 min (approximately 40 min for most interviews). After each interview, community researchers had a debrief meeting with their paired researcher to reflect on the experience, and to discuss any issues or concerns.

### 2.7 Analysis

Audio recordings were transcribed verbatim. Two interviews conducted in Arabic were translated and transcribed into English by a member of the research support team. Our approach to analysis was inductive using reflexive thematic analysis [38, 39]. Five researchers brought ideas to the early analytical process (GL, OJ, AA, RC, RH) by independently familiarising themselves with the transcripts, noting initial impressions for each transcript and highlighting key extracts. Following this, researchers engaged in group discussions to share analytic reflections framed by the research questions. These discussions formed the basis of an initial coding framework. A single researcher (GL) reviewed and coded all transcripts using an iterative approach to further develop the coding framework. Theme generation was an interpretive, reflexive process carried out collaboratively by two researchers (GL, OJ), in line with reflexive thematic analysis, revisiting the raw data for all transcripts throughout this process. The preliminary findings were shared with the wider team for discussion. The community researcher team members (NA, LB, AK, SK) discussed the findings with GL or OJ to deepen interpretation and enrich analytic insights. GL and OJ engaged in further discussions to refine the final themes. The Reflexive Thematic Analysis Reporting Guidelines were followed to enhance the quality and trustworthiness of the study [40].

### 2.8 Findings

**Figure 1:**
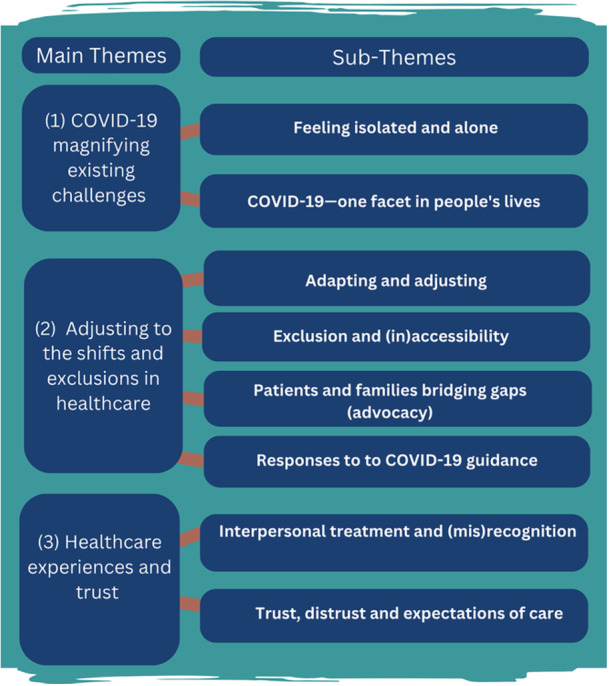
Thematic diagram.

We present three themes, each with its own subthemes: (1) COVID‐19 magnifying existing challenges; (2) Adjusting to the shifts and exclusions in healthcare, and (3) Healthcare experiences and trust (see Figure 1 for a thematic diagram). These themes were evident across participants, however where appropriate, specific issues and examples that were more salient within specific communities of interest are highlighted.

### 2.9 Theme 1: COVID‐19 Magnifying Existing Challenges

The first theme contains two subthemes which explore feelings of isolation and loneliness and managing multiple challenges brought on by competing demands. Throughout the COVID‐19 pandemic, many participants grappled with profound psychological and social impacts. Simultaneously, they navigated a multitude of pressing life priorities amidst the pandemic's disruptions, often struggling to ensure their families’ basic needs and life necessities were met. Despite varying levels of social connectivity, participants expressed that the pandemic compounded existing life challenges.

#### 2.9.1 Subtheme 1a: Feeling Isolated and Alone

People talked about experiencing poorer mental health and the realities of the isolation they experienced, and the profound impact of feeling disconnected. People were significantly affected by not attending their usual community activities and groups. Most participants described feeling a loss of regular social interaction, loss of structure and purpose in life and without the structure that helped them manage their wellbeing. For some, these groups were their primary source of community engagement, which contributed to worsening mental health. One person described how the closure of community centres intensified her sense of loneliness:

…all that got taken away as well… it was like oh my God it feels like…I'm totally on my own I'm a prisoner in this, it was awful. Mentally, it really really got to me.

(Organisation 1, ID2)

For others, particularly recent migrants, the pandemic compounded an existing sense of dislocation. They explained how restrictions amplified the challenges of being new to the UK:

…because I'm stressed all the time, I'm thinking about my kids to um, to protect them… I didn't go outside a lot, I didn't talk a lot this time cause you're not allowed to meet any people, I feel I'm alone in this country, especially in the beginning and, like you don't feel comfortable, you don't feel err, you want to speak you can't speak if you want to see another people. You can't. You can't do anything you should, you have to stay at home…

(Organisation 2‐4, ID5)

The inability to visit friends or extended family deepened this isolation:

Yes, you can't see anyone, you can't be with anyone, you can't see friends you can't visit anyone in their house.

(Organisation 2‐4, ID24)

Even though people faced significant challenges during this time, there was also a sense of people ‘making the best of it’.

#### 2.9.2 Subtheme 1b: COVID‐19 as One Facet in People's Lives

The COVID‐19 pandemic was not always the most significant event or issue in people's lives. People described navigating a broader set of overwhelming life demands. In our data, unstable living situations, moving from another country, financial instability, uncertainty around job prospects and exams, were all described as salient challenges alongside the pandemic.

For people seeking asylum, unresolved immigration cases dominated their emotional and cognitive load. One participant described repeated, unsuccessful attempts to contact the Home Office for support during lockdown:

I think during like lockdown because you know Home Office was in our neck and we couldn't do anything we had to write a lot of letters for us to like find help what can we do we will try to write words…… no reply, no response. We tried almost everything. We almost gave up……

(Organisation 2‐4, ID13)

Among young people, educational uncertainty and disrupted routines generated significant stress:

Probably the third lockdown in January this year. That was probably the height of like the worst part for me personally, like I wasn't seeing anyone. My mental health was lower and like it was affecting my exams and stuff and in like college, so that was all a bit stressful…. there was like online work but it wasn't easy to access like and…I felt like I wasn't getting anything done

(Organisation 1, ID20)

Additionally, housing instability and financial stress often overshadowed the pandemic itself:

…the thing we were going through the thing my family was going through…It was we were struggling with keeping a roof over our head with paying the rent. We were almost being thrown out from the house, so you know everything was accumulating so much in the pandemic. I mean that it was like, you know, it was not the COVID, it was just. It looked like it was like the end of the world for us in that time…

(Organisation 2‐4, ID14)

More specifically, people seeking asylum tended to experience acute insecurity tied to their unresolved immigration processes, whereas participants with refugee status faced ongoing resettlement and post‐migration pressures that were further amplified by COVID‐19 disruptions.

### 2.10 Theme 2: Adjusting to the Shifts and Exclusions in Healthcare

The second theme highlights that participants followed protective actions to protect themselves against COVID‐19, despite at times, perceptions of unclear and inadequate Government public health messaging. Participants described a sense of personal, familial and social responsibility to safeguard themselves and others, however, in certain circumstances, access to protective equipment and facilities were extremely challenging. Participants tried and learned new approaches to navigate the healthcare system, constantly adapting in the face of adversity.

#### 2.10.1 Subtheme 2a: Adapting and Adjusting

Participants shared needing to continually adapt to rapidly changing health requirements and new modes of accessing healthcare during the pandemic. The evolving public health measures and system‐level changes meant that people were repeatedly required to adjust how they sought care, communicated with services, and navigated access pathways. This involved shifts to new processes, such as remote consultations, digital triage systems, online forms and altered testing procedures. These shifts required participants to modify how they had learned to engage with services, often under conditions of uncertainty and limited support.

For some, this shift required developing unfamiliar digital skills, which created the practical burden of repeated attempts to access:

…trying to send a picture in the first place was difficult because I've never done that before so they send you a link and then you've got to try and go onto it, click onto it and then you've got to add your few details and send the picture, which is all quite new to me…

(Organisation 1, ID1)

Others described the physical hassle of unclear and inconsistent pathways before securing the tests they required:

….I had to go through a lot for me to get the first test because when I did send it back, it came back without a decision, it couldn't tell. So then I had to, I booked an appointment and I went all the way somewhere and they said they couldn't test him. So it was a big hassle, and then finally I went to [name of an organisation] and they tested him there

(Organisation 1, ID3)

Participants also spoke about the loss of direct communication with healthcare professionals and the difficulties this posed for explaining symptoms or accessing medication:

Yeah, it's not possible to communicate with a doctor face to face because of corona barriers. So, we have a problem but I understand that but it's more for the patient problem for the home screen problem that is not available to conversation by medication situation everything is not good

(Organisation 2‐4, ID15)

#### 2.10.2 Subtheme 2b: Exclusion and (In)Accessibility

There were numerous issues raised regarding the inaccessibility of healthcare services at this time. Many people found the COVID‐19 testing process to be unnecessarily complicated. Healthcare guidance and communication was often not in an accessible format that catered to people's different needs e.g. for cultural differences, language, and learning disabilities. People frequently encountered systems designed with assumptions about digital literacy, language fluency and access to resources such as internet, devices and transport.

Some participants described the increased digital demands as overwhelming with high technological knowledge and skill required for registering, completing forms and returning information. One participant recalls the difficulty of accessing COVID‐19 testing:

Trust me you need an education for it I've heard like my mum would never be able to, they are making it very difficult for people…You have to register about three different numbers, you have to know where you get your registration number from…Once you even managed to figure out where you supposed to post it, you have to travel somewhere to post it…

(Organisation 1, ID3)

One participant described the practical challenges for both the participant and healthcare staff of using technology to send clinical photographs, as staff were unable to adequately assess the issue and required an in‐person visit:

…Yeah I've never done it before … and it was like hard taking the pictures and I didn't know how to do it and I did manage to send them, once she had them, she l‐ she said look… I've got the pictures, but I rather you come down and I wanna see it myself… I need to have a look at it. So even on the pictures she couldn't tell…

(Organisation 1, ID2)

Language barriers further intensified exclusion. Participants with limited English shared feeling uncomfortable, anxious and at times unable to communicate their health needs:

…Yes, the first things for anyone who don't speak English or weak English, you don't feel comfortable in speak, you shy, especially in the beginning if there's interpreter in the pharmacy or in the GP that will be better for the family, feel comfortable to talk with, happy to talk, but without‐ without English, very, very difficult for everything…

(Organisation 2‐4, ID5)

Participants living in unstable housing situations perceived their environment and resources available as common barriers to protective health behaviours:

…I remember we had in, in this place, they have common areas, the people didn't use masks, no distance people, I remember that one family have COVID, 1 day ago, this family was in the common areas with other people without masks. Yes, and they ‐ they never, the next day, this family, was in quarantine 15 days but, the old people was in this area…

(Organisation 2‐4, ID23 ‐ discussing temporary accommodation)

The shift toward digital, remote and rapidly changing procedures such as social distancing, handwashing and COVID‐19 testing, inadvertently excluded those already marginalised and added additional burden to navigate the system.

#### 2.10.3 Subtheme 2c: Patients and Families Bridging Gaps

People talked about their commitment to behaviours that protected themselves and others (e.g. hand washing, social distancing, wearing a mask). Participants frequently relied on family members to interpret, advocate and navigate procedures, which involved considerable emotional and logistical work.

There were examples of family members supporting and advocating for their loved ones, managing multiple responsibilities and coordinating others’ care. For example, one participant relied on her son to communicate with the GP:

My son helped me…because sometimes I don't understand… all the time I say that…My comprehension, n‐not very well

(Organisation 2‐4, ID11 ‐ discussing whether it is easy to call the GP)

Others advocated persistently for their children's care during moments of acute episodes, sometimes with the need to travel ‘to go really far for surgery’, whilst others negotiated multiple healthcare settings:

… my daughter had taken like really bad, we had to rush to A&E and she just got a very high grade fever… I gave the GP a call in what exactly happened is that I requested A&E to do a test for her with COVID… I was begging for my child to be seen because I know from the time she's born till now she's never had this grade fever…

(Organisation 2‐4, ID14 ‐ advocating for a child with a fever, moved between A&E, GP and Pharmacy)

Families were often providing support for their loved ones in an overstretched system, working around structural barriers to secure the care they required.

#### 2.10.4 Subtheme 2d: Responses to COVID‐19 Guidance

People described the challenge of encountering conflicting information from different sources, creating uncertainty about what actions to take. Official reports were received alongside community messaging. People shared different approaches to seeking information, some people did not want to take in information to protect their mental health citing ‘information overload’, while others actively stayed informed by cross‐checking government websites and trusted online sources.

Several people explained conflicting reports and information about vaccines:

To be honest, there were conflicting news reports coming out like on‐ like on Whatsapp groups or on messages people send you, they'd say things like umm, like umm what they called, electric pylons…

(Organisation 1, ID1)

Participants seeking clarity checked official websites:

…I always, search information in the government page, yes

(Organisation 2‐4, ID23)

However, the official guidance was confusing due to constant changes and rules in different sectors:

…because it wasn't clear when they'd give you the information and then when I think everybody started to understand what it was they change it and say well we're gonna change it to a tiering system or red amber green, or that, you know you can meet in families of 10, but it has to be outdoors but it, it was just confusing to keep up to date with all of it…with universities and with jobs it was difficult because everybody had different rules sometimes, so it was hard

(Organisation 1, ID16)

For those with precarious immigration status, compliance carried additional emotional weight. One participant feared the consequences of being seen as non‐compliant:

…Who am I not to say no? But whatever advice the government brings, we just go with it. Because if you don't, that's going to be recorded against you as being insubordinate. You are not obeying the laws; you're not adhering to the rules

(Organisation 2‐4, ID12)

Challenges with COVID‐19 were not only about information clarity or accessibility but shaped by structural inequities and heightened vulnerabilities experienced by people's social position, which intensified the perceived consequences of taking the 'wrong' actions.

### 2.11 Theme 3: Healthcare Experiences and Trust

Participants’ healthcare experiences during the COVID‐19 pandemic were often interpreted through the lens of earlier encounters, influencing expectations about whether they would be treated with respect or dismissed. Participants’ trust in healthcare were shaped by both interpersonal interactions and their broader social positions, particularly individuals with intersecting identities (e.g. racialised and gendered) with examples of negative, dismissive or discriminatory interactions. These gaps in support were often ‘bridged’ through the advocacy of family and community members. Many participants described the challenge of navigating uncertainty around COVID‐19 guidance and COVID‐19 information sources.

#### 2.11.1 Subtheme 3a: Interpersonal Treatment and (Mis)Recognition

Participants’ accounts of healthcare were shaped not only by their experiences during the COVID‑19 pandemic, but also by earlier encounters that informed how they interpreted, trusted, and engaged with services once the pandemic began. Many participants acknowledged the intense pressure on healthcare systems and recognised the challenges faced by staff. There were examples of negative healthcare experiences, including rudeness; negative attitudes; cultural assumptions; dismissed medical concerns; and experiences of discrimination. Positive experiences were evident, but limited.

Several examples of distressing or confusing interactions were shared, as illustrated by one participant who recalled being laughed at by a staff member due to language difficulties:

Because sometimes I don't understand, my comprehension I know is bad, my English I try, all the time to learn, more and more practice my English, but I don't know why the girl laughed and told them, I went to my second destination

(Organisation 2‐4, ID11)

People described feeling misunderstood or judged because of their circumstances, with examples of bias and cultural insensitivity. The following quote from a South Asian parent assumed to have social support, despite having no childcare options, illustrates this experience:

I had to bring her [daughter] and they and they were not happy about it, but I said, look I've got nobody else, and they were like oh you must've got somebody who could've looked [after her]….

(Organisation 1, ID2)

Further, participants described negative experiences influenced by social identity, including feeling scrutinised or devalued with concerns not taken seriously:

….I wasn't happy for the fact that she mentioned that I am on support........that I am here to waste their resources, or I should be grateful or I should. I should believe that nothing is to be done. I should wait for them until at least I should have a say.

(Organisation 2‐4, ID12)

#### 2.11.2 Subtheme 3b: Trust, Distrust and Expectations of Care

Participants’ accounts reflected complex and often shifting experiences of trust during the COVID‐19 pandemic. Despite challenges, a limited number of participants described positive healthcare experiences during the pandemic that strengthened their trust through compassionate, personalised and relational care. For example, one participant illustrated how their GP recognised their emotional difficulties during the pandemic and facilitated access to counselling and signposting to community support services:

…I was recommended for the counselling by the GP. He said like, you know, you definitely need counselling… And they helped me. And its not only the counselling help me, that did help me a lot, but I was still a bit on medication. But what they did is they gave me some organisation where I could call and ask them for help…

(Organisation 2‐4, ID15)

However, trust was not fixed or straightforward and was negotiated amidst uncertainty, contradictory information and rapidly changing public health guidance. The confusion and inconsistency in government messaging described in Subtheme 2d, contributed to scepticism, undermining confidence in institutions and official guidance. One participant explained:

… they say don't go to school, but then go to pubs…it doesn't make sense…so then people think… is this coronavirus really real

(Organisation 2–4, ID3)

For some participants, trust was selective and conditional shaped by perceptions of credibility, familiarity and lived experience. For example, one participant described how they only trusted information from the NHS and not the community, but their understanding of COVID‐19 shifted after experiencing the illness personally:

…when I had it I can now believe anyone to be honest ‘cause I know COVID is very tired, pain, stressful.

(Organisation 2‐4, ID9)

For many participants, trust remained fragile and conditional, shaped by cumulative experiences of exclusion, dismissal and unequal treatment that were intensified during the pandemic. Several participants described feeling unable to challenge decisions or raise concerns because they felt their voices would not be heard or taken seriously. This sense of powerlessness contributed to feelings of distrust towards healthcare systems and professionals. One participant recently seeking asylum reflected:

…there is nothing I'm going to bring to complain about your system, so I just have to take the injustice. You won't listen to me in the first place, so its of no use…the confidence and trust is not there…

(Organisation 2‐4, ID12)

These participants felt unsupported and unheard with cumulative distrust, contributing to perceptions of the absence of meaningful responses from healthcare systems.

## 3 Discussion

Our research adopted a community research approach to understand how women from South Asian backgrounds and people seeking asylum and refuge accessed healthcare and looked after their health and the health of others during the COVID‐19 pandemic.

For these marginalised groups, poor healthcare experiences and issues relating to healthcare access were well evidenced prior to the COVID‐19 pandemic, including discrimination, cultural and linguistic exclusion and fragmented access to care [41, 42, 43, 44, 45]. Our research demonstrates that these existing healthcare inequalities and inequities were often compounded during the pandemic.

Previous research acknowledges that patients and families can support the quality and safety of care [46] and the resilience of healthcare services and systems during crises [47]. In our research, there were multiple examples of patients and families consistently reaching into services to support the quality and safety of care. Despite this, poor healthcare experiences were still prominent for many participants, indicating that the issues extend beyond a simple need for patients or families to ‘reach in’. These experiences may reflect more fundamental structural inequities within the healthcare system, including cultural and linguistic exclusion, discriminatory interpersonal treatment, digital barriers and system level processes that do not account for the needs of racialised, migrant or socio‐economically marginalised groups.

Consistent with wider literature, participants highlighted compounding challenges created by government policies, limited access to protective resources, negative experiences with the healthcare service and a perception of conflicting government guidance [16, 48]. For people seeking asylum and refuge, unsafe conditions contributed to difficulties adhering to public health guidance due to inadequate access to basic resources such as private spaces, washing facilities, adequate space to distance or relational support (e.g. health service restrictions of visitation to the hospital for people with multiple dependents and no social support in the country). Despite these barriers, it was evident that there was approval of and an attempt to adhere to the government policies and guidelines, reflecting a strong sense of personal and collective responsibility. Efforts were shaped by structural constraints that limited the extent to which people felt they were able to comply with guidance as well as their confidence in doing so. Specifically for people seeking asylum, this often included fearing the consequences of non‐compliance due to the precarity of their status.

Our findings reinforce longstanding documentation of stigma, discrimination and prejudice experienced by people seeking asylum and refugees within healthcare settings prior to ([45, 49], p22) and during the COVID‐19 pandemic [16]. These systemic barriers were demonstrated by healthcare responses which often failed to meet the needs of diverse populations, especially those who spoke English as an additional language or lacked digital access. Understanding and intentionally addressing these experiences is vital, as negative healthcare encounters can contribute to long term erosion of confidence and trust in healthcare services and a reluctance to escalate healthcare concerns ([49], p40).

### 3.1 Implications and Recommendations

Our research aimed to generate evidence and recommendations for policy makers, healthcare systems and the healthcare workforce in the context of the COVID‐19 pandemic response, to inform future pandemic responses. The COVID‐19 pandemic exacerbated pre‐existing challenges of exclusion faced by patients, carers, and the wider public in health service decision‐making [11, 18].

### 3.2 For Policy and Policy Making

Our findings illustrate the importance of an intentional inclusion of marginalised experiences, ideas and perspectives of underrecognised groups in policy development, service design and research [43, 50]. This proactive approach should be embedded systematically rather than activated reactively in emergencies, as it helps to redress the imbalance of perspectives and avoids excluding those that are often disproportionately harmed. To enhance public health messaging and the importance of safety, it is essential to develop clear, relatable, and culturally tailored communications that address societal diversity. These should be co‐produced with communities and disseminated through localised communication strategies. Working with trusted sources in the community will encourage protective behaviours and engagement with different health measures, as well as counter misinformation campaigns [51].

Resource allocation must reflect the needs of marginalised groups with an emphasis on redistributing funding to community organisations. Their active role in decision making will enable the coproduction of services and policies that are responsive and equitable. Building and maintaining trust‐based collaborations with communities requires ongoing investment in infrastructure to leverage local strengths, skills, and resources.

### 3.3 For Practice

Future pandemic planning must prioritise equitable resource allocation (e.g. protective resources) and recognise the specific challenges faced by people in overcrowded facilities and limited access to private spaces [16, 48]. It is important for healthcare services to strengthen and enforce anti‐discrimination policies for all healthcare staff to build awareness and understanding of cultural, and linguistic diversity, including knowledge of migrant populations, emphasising the harmful impact of biases and stereotypes on patient care.

Cultural humility refers to the importance of having a respectful and open attitude towards other cultures, engaging in continuous self‐reflection to challenge personal and collective biases, and an ongoing approach to learning about other cultures [29, 43]. Organisations and individuals should promote, enshrine and evaluate cultural humility [43] as a core element of their processes, practices and clinical interactions. Our findings point to concrete actions relevant to the specific communities in this study. For South Asian women this may include improving culturally responsive communication, accessible interpreting services, and flexible appointment models, that recognise those with childcare responsibilities and gendered expectations. For people seeking asylum, priority actions include tailored public health communication that accounts for language barriers, alternative measures for those in unstable living conditions and the fears associated with insecure immigration status. Additionally, for people with refugee status, it is important to include non‐digital routes to healthcare access e.g. in‐person booking, walk‐in options and strengthening partnerships with community organisations with expertise, to provide trusted, culturally informed support.

### 3.4 For Research

Our asset‐based approach highlighted the value of a diverse, collaborative and multidisciplinary team which leveraged community knowledge with research expertise. For example, two interviews were conducted in Arabic, and transcribed by a member of our research team, which minimised exclusion based on language. This innovative participatory approach privileged the experiences of marginalised communities and future community research with marginalised communities should endeavour to identify the ‘gaps’, ‘traps’, ‘bridges’ and ‘props’ for upholding healthcare safety beyond the COVID‐19 context [52]. Whilst there were many benefits of working in a large team, the fluctuating nature of employment presented a limitation within both the voluntary, community and social enterprises (VCSE) and the research sector, which provided valuable learning for future research. Pairing community researchers with multiple link researchers from healthcare research organisations may help ensure continuity and support despite staff turnover.

## 4 Conclusion

Despite the significant and compounding challenges woman from South Asian backgrounds and people seeking asylum and refugees experienced during the COVID‐19 pandemic, participants demonstrated resilience and adaptability in navigating the healthcare system and managing protective behaviours. With growing diversity within the population, healthcare services must be adaptable to meet different needs and deliver safe, high‐quality care for all. By embracing participatory approaches, strengthening collaboration with community organisations, and intentionally accounting for structural inequities, health systems can enhance preparedness for future public health emergencies and move towards more inclusive, equitable care.

## Author Contributions

**Olivia Joseph:** conceptualisation, methodology, formal analysis, supervision, project administration, writing – original draft, writing – review and editing. **Nadia Ait Yahya:** investigation, formal analysis, project administration, writing – review and editing. **Lilly Butt:** investigation, formal analysis, project administration, writing – review and editing. **Amana Khan:** investigation, formal analysis, project administration, writing – review and editing. **Sabiya Khan:** investigation, formal analysis, project administration, writing – review and editing. **Jane K. O'Hara:** conceptualisation, methodology, writing – review and editing. **Abigail Albutt:** conceptualisation, methodology, formal analysis, writing – review and editing. **Ryan Carter:** formal analysis, writing – review and editing. **Rameen Haq:** formal analysis, writing – review and editing. **Gemma Louch:** conceptualisation, methodology, formal analysis, project administration, supervision, writing – original draft, writing – review and editing.

## Ethics Statement

Ethical approval for this study was granted by the University of Leeds, School of Medicine Research Ethics Committee (SoMREC), reference MREC 19‐076.

## Conflicts of Interest

The authors declare no conflicts of interest.

## Supporting information

Supporting File

## Data Availability

Research data are not shared. All requests relating to accessing data and/or materials should be submitted to the corresponding author and will be considered on a case‐by‐case basis.

## Endnotes

1 Zoom is an encrypted secure video conferencing software platform.

## References

[1] H. Alizadeh , A. Sharifi , S. Damanbagh , H. Nazarnia , and M. Nazarnia , “Impacts of the COVID‐19 Pandemic on the Social Sphere and Lessons for Crisis Management: A Literature Review,” Natural Hazards 117, no. 3 (July 2023): 2139–2164. 10.1007/s11069-023-05959-2 PMC10088618 37360799

[2] I. Chakraborty and P. Maity , “COVID‐19 Outbreak: Migration, Effects on Society, Global Environment and Prevention,” Science of the Total Environment 728 (August 2020): 138882. 32335410 10.1016/j.scitotenv.2020.138882 PMC7175860

[3] O. Fersia , S. Bryant , R. Nicholson , et al., “The Impact of the COVID‐19 Pandemic on Cardiology Services,” Open Heart 7, no. 2 (August 2020): e001359. 32855212 10.1136/openhrt-2020-001359 PMC7454176

[4] R. Moynihan , S. Sanders , Z. A. Michaleff , et al., “Impact of COVID‐19 Pandemic on Utilisation of Healthcare Services: A Systematic Review,” BMJ Open 11, no. 3 (March 2021): e045343. 10.1136/bmjopen-2020-045343 PMC7969768 33727273

[5] G. Pujolar , A. Oliver‐Anglès , I. Vargas , and M. L. Vázquez , “Changes in Access to Health Services During the COVID‐19 Pandemic: A Scoping Review,” International Journal of Environmental Research and Public Health 19, no. 3 (February 2022): 1749. 35162772 10.3390/ijerph19031749 PMC8834942

[6] L. L. Gleeson , A. Ludlow , E. Wallace , et al., “Changes to Primary Care Delivery During the COVID‐19 Pandemic and Perceived Impact on Medication Safety: A Survey Study,” Exploratory Research in Clinical and Social Pharmacy 6 (June 2022): 100143. 35702683 10.1016/j.rcsop.2022.100143 PMC9182324

[7] R. Lewis , P. Pereira , R. Thorlby , and W. Warburton , Understanding and Sustaining the Health Care Service Shifts Accelerated By COVID‐19 (The Health Foundation, 2020).

[8] K. Søreide , J. Hallet , J. B. Matthews , et al., “Immediate and Long‐Term Impact of the COVID‐19 Pandemic on Delivery of Surgical Services,” British Journal of Surgery 107, no. 10 (September 2020): 1250–1261. 32350857 10.1002/bjs.11670 PMC7267363

[9] C. Zangani , E. G. Ostinelli , K. A. Smith , et al., “Impact of the COVID‐19 Pandemic on the Global Delivery of Mental Health Services and Telemental Health: Systematic Review,” JMIR Mental Health 9, no. 8 (August 2022): e38600. 35994310 10.2196/38600 PMC9400843

[10] S. Ali , M. Asaria , and S. Stranges , “COVID‐19 and Inequality: Are We All in This Together?,” Canadian Journal of Public Health 111 (June 2020): 415–416. 32578185 10.17269/s41997-020-00351-0 PMC7310590

[11] S. Bhaskar , A. Rastogi , K. V. Menon , B. Kunheri , S. Balakrishnan , and J. Howick , “Call for Action to Address Equity and Justice Divide During Covid‐19,” Frontiers in Psychiatry 11 (December 2020): 559905. 33343410 10.3389/fpsyt.2020.559905 PMC7744756

[12] S. V. Katikireddi , S. Lal , E. D. Carrol , et al., “Unequal Impact of the COVID‐19 Crisis on Minority Ethnic Groups: A Framework for Understanding and Addressing Inequalities,” Journal of Epidemiology and Community Health 75, no. 10 (October 2021): 970–974. 33883198 10.1136/jech-2020-216061 PMC8458062

[13] J. E. Madia , C. Nicodemo , and S. Redding , “Ethnicity and Inequality during the COVID‐19 Pandemic in the UK.” Economics of COVID‐19 (Emerald Publishing Limited, 2022), 296, 143–158.

[14] Z. Mengesha , E. Alloun , D. Weber , M. Smith , and P. Harris , “Lived the Pandemic Twice: a Scoping Review of the Unequal Impact of the COVID‐19 Pandemic on Asylum Seekers and Undocumented Migrants,” International Journal of Environmental Research and Public Health 19, no. 11 (May 2022): 6624. 35682211 10.3390/ijerph19116624 PMC9180209

[15] V. Mishra , G. Seyedzenouzi , A. Almohtadi , et al., “Health Inequalities During COVID‐19 and Their Effects on Morbidity and Mortality,” Journal of Healthcare Leadership 13 (January 2021): 19–26. 33500676 10.2147/JHL.S270175 PMC7826045

[16] M. Nouri , A. Ostadtaghizadeh , and A. A. Sari , “COVID‐19 in Homelessness: A Worldwide Scoping Review on Vulnerabilities, Risks, and Risk Management,” Social Work in Public Health 37, no. 4 (May 2022): 303–318. 34963409 10.1080/19371918.2021.2011525

[17] A. Paton , G. Fooks , G. Maestri , and P. Lowe Submission of Evidence on the Disproportionate Impact of COVID 19, and the UK Government Response, on Ethnic Minorities and Women in the UK.

[18] N. M. Rodriguez , R. G. Martinez , R. Ziolkowski , C. Tolliver , H. Young , and Y. Ruiz , “‘Covid Knocked Me Straight Into the Dirt’: Perspectives From People Experiencing Homelessness on the Impacts of the COVID‐19 Pandemic,” BMC Public Health 22, no. 1 (Jul 2022): 1327. 35820879 10.1186/s12889-022-13748-y PMC9275174

[19] A. J. Stevens , A. M. Ray , A. Thirunavukarasu , et al., “The Experiences of Socially Vulnerable Groups in England During the COVID‐19 Pandemic: A Rapid Health Needs Assessment,” Public Health in Practice 2 (November 2021): 100192. 34608460 10.1016/j.puhip.2021.100192 PMC8481647

[20] S. E. Hayward , A. Deal , C. Cheng , et al., “Clinical Outcomes and Risk Factors for COVID‐19 Among Migrant Populations in High‐Income Countries: A Systematic Review,” Journal of Migration and health 3: 100041. 10.1016/j.jmh.2021.100041 PMC8061095 33903857

[21] N. M. Lou , K. A. Noels , Y. S. D. Zhang , and S. Kurl , “Ethnic Minority, Immigrants, and Indigenous People's Well‐Being Disparities in Canada During the COVID‐19 Pandemic: The Mediating Role of Threat Perceptions,” International Journal of Intercultural Relations 88 (2022): 148–156, 10.1016/j.ijintrel.2022.04.006. 35475126 PMC9023321

[22] A. Yong and S. Germain , “Ethnic Minority and Migrant Women's Struggles in Accessing Healthcare During COVID‐19: An Intersectional Analysis,” Journal for Cultural Research 26, no. 1 (January 2022): 65–82.

[23] F. Knights , J. Carter , A. Deal , et al., “Impact of COVID‐19 on Migrants’ Access to Primary Care and Implications for Vaccine Roll‐Out: A National Qualitative Study,” British Journal of General Practice 71, no. 709 (August 2021): e583–e595. 10.3399/BJGP.2021.0028 PMC8216266 33875420

[24] I. Adeyemi , C. Sanders , B. N. Ong , et al., “Challenges and Adaptations to Public Involvement With Marginalised Groups During the COVID‐19 Pandemic: Commentary With Illustrative Case Studies in the Context of Patient Safety Research,” Research Involvement and Engagement 8, no. 1 (April 2022): 13. 35410450 10.1186/s40900-022-00345-x PMC8996501

[25] J. Ocloo , “Being Heard, Not,‘Seldom Heard’: Democratising Research With Diverse Communities During the covid‐19 Pandemic,” BMJ Opinion 2 (June 2020), https://blogs.bmj.com/bmj/2020/06/02/being-heard-not-seldom-heard-democratising-research-with-diverse-communities-during-the-covid-19-pandemic/.

[26] J. Salma and D. Giri , “Engaging Immigrant and Racialized Communities in Community‐Based Participatory Research During the COVID‐19 Pandemic: Challenges and Opportunities,” International Journal of Qualitative Methods 20 (August 2021): 16094069211036293.

[27] K. B. Wells , F. Jones , and K. C. Norris , “Applying Community‐Partnered Participatory Research Approaches to Develop COVID‐19 Solutions,” Ethnicity & Disease 30, no. 3 (2020): 433–436. 32742147 10.18865/ed.30.3.433 PMC7360174

[28] Y. Suarez‐Balcazar , V. T. Francisco , and N. Rubén Chávez , “Applying Community‐Based Participatory Approaches to Addressing Health Disparities and Promoting Health Equity,” American Journal of Community Psychology 66, no. 3/4 (December 2020): 217–221. 33373469 10.1002/ajcp.12487

[29] R. Robertson , E. Williams , D. Buck , and J. Breckwoldt, Ethnic Health Inequalities and the NHS. In NHS Race and Health Observatory & The King's Fund, Ethnic Health Inequalities, 2021, https://www.nhsrho.org/wp-content/uploads/2023/05/Ethnic-Health-Inequalities-Kings-Fund-Report.pdf.

[30] World Health Organization , 2020, *COVID‐19 Global Risk Communication and Community Engagement Strategy*: *December 2020–May 2021: Interim Gguidance* , World Health Organization, Accessed 20/05/2026, https://www.who.int/publications/i/item/covid-19-global-risk-communication-and-community-engagement-strategy.

[31] L. Goodson and J. Phillimore , “A Community Research Methodology: Working With New Migrants to Develop a Policy Related Evidence Base,” Social Policy and Society 9, no. 4 (October 2010): 489–501.

[32] N. B. Wallerstein and B. Duran , “Using Community‐Based Participatory Research to Address Health Disparities,” Health Promotion Practice 7, no. 3 (July 2006): 312–323. 16760238 10.1177/1524839906289376

[33] J. Daly Lynn , M. Washbrook , A. Ryan , B. McCormack , and S. Martin , “Partnering With Older People as Peer Researchers,” Health Expectations 24, no. 5 (October 2021): 1879–1889. 34337838 10.1111/hex.13331 PMC8483193

[34] K. Devotta , J. Woodhall‐Melnik , C. Pedersen , et al., “Enriching Qualitative Research by Engaging Peer Interviewers: A Case Study,” Qualitative Research 16, no. 6 (December 2016): 661–680.

[35] S. Reeves , M. Albert , A. Kuper , and B. D. Hodges , “Why Use Theories in Qualitative Research?,” BMJ 337 (August 2008): a949. 18687730 10.1136/bmj.a949

[36] P. Braveman , E. Arkin , T. Orleans , D. Proctor , J. Acker , and A. Plough , “What Is Health Equity?,” Behavioral Science & Policy 4, no. 1 (April 2018): 1–4.

[37] K. Malterud , V. D. Siersma , and A. D. Guassora , “Sample Size in Qualitative Interview Studies: Guided by Information Power,” Qualitative Health Research 26, no. 13 (November 2016): 1753–1760. 26613970 10.1177/1049732315617444

[38] V. Braun and V. Clarke , “Using Thematic Analysis in Psychology,” Qualitative Research in Psychology 3, no. 2 (2006): 77–101, 10.1191/1478088706qp063oa.

[39] V. Braun and V. Clarke , “Reflecting on Reflexive Thematic Analysis,” Qualitative Research in Sport Exercise and Health 11, no. 4 (2019): 589–597, 10.1080/2159676x.2019.1628806.

[40] V. Braun and V. Clarke , “Supporting Best Practice in Reflexive Thematic Analysis Reporting in Palliative Medicine: A Review of Published Research and Introduction to the Reflexive Thematic Analysis Reporting Guidelines (Rtarg),” Palliative Medicine 38, no. 6 (2024): 608–616, 10.1177/02692163241234800. 38469804 PMC11157981

[41] C. Kang , L. Tomkow , and R. Farrington , “Access to Primary Health Care for Asylum Seekers and Refugees: A Qualitative Study of Service User Experiences in the Uk,” British Journal of General Practice 69, no. 685 (August 2019): e537–e545. 10.3399/bjgp19X701309 PMC6617541 30745354

[42] T. Pollard and N. Howard , “Mental Healthcare for Asylum‐Seekers and Refugees Residing in the United Kingdom: A Scoping Review of Policies, Barriers, and Enablers,” International Journal of Mental Health Systems 15, no. 1 (June 2021): 60. 34127043 10.1186/s13033-021-00473-z PMC8201739

[43] A. Sayani , A. Maybee , J. Manthorne , et al., “Building Equitable Patient Partnerships During the COVID‐19 Pandemic: Challenges and Key Considerations for Research and Policy,” Healthcare Policy | Politiques de Santé 17, no. 1 (2021): 17–24, 10.12927/hcpol.2021.26582. 34543172 PMC8437252

[44] Szczepura A. “Access to Health Care for Ethnic Minority Populations,” Postgraduate Medical Journal 81, no. 953 141–147. 15749788 10.1136/pgmj.2004.026237 PMC1743229

[45] K. Taylor , “Asylum Seekers, Refugees, and the Politics of Access to Health Care: a UK Perspective.” In Health and Human Rights in a Changing World (Routledge, 2013), 289–300.

[46] J. K. O'Hara , K. Aase , and J. Waring , “Scaffolding Our Systems? Patients and Families ‘Reaching In'as a Source of Healthcare Resilience,” BMJ Quality & Safety 28, no. 1 (January 2019): 3–6. 10.1136/bmjqs-2018-008216 29764929

[47] A. Albutt , L. Ramsey , B. Fylan , C. Grindey , I. Hague , and J. K. O'Hara , “Patient and Public Co‐Creation of Healthcare Safety and Healthcare System Resilience: The Case of COVID‐19,” Health Expectations 26, no. 4 (August 2023): 1467–1477. 37139679 10.1111/hex.13659 PMC10349237

[48] Z. Asif and H. Kienzler , “Structural Barriers to Refugee, Asylum Seeker and Undocumented Migrant Healthcare Access. Perceptions of Doctors of the World Caseworkers in the Uk,” SSM ‐ Mental Health 2 (2022): 100088, 10.1016/j.ssmmh.2022.100088.

[49] Equality and Human Rights Commission , Nellums, L. B., Rustage, K., Hargreaves, S., Friedland, J. S., Imperial College London, Miller, A., Hiam, L., & Doctors of the World UK. (2018). The Lived Experiences of Access to Healthcare for People Seeking and Refused Asylum, https://www.equalityhumanrights.com/sites/default/files/2022/our-work-research-people-seeking-asylum-access-healthcare-lived-experiences-2018.pdf.

[50] P. Cairney and A. Wellstead , “COVID‐19: Effective Policymaking Depends on Trust in Experts, Politicians, and the Public,” Policy Design and Practice 4, no. 1 (2021): 1–14, 10.1080/25741292.2020.1837466.

[51] B. Lockyer , S. Islam , A. Rahman , et al., “Understanding COVID‐19 Misinformation and Vaccine Hesitancy in Context: Findings From a Qualitative Study Involving Citizens in Bradford, UK,” Health Expectations 24, no. 4 (2021): 1158–1167, 10.1111/hex.13240. 33942948 PMC8239544

[52] B. Fylan , I. Marques , H. Ismail , et al., “Gaps, Traps, Bridges and Props: A Mixed‐Methods Study of Resilience in the Medicines Management System for Patients With Heart Failure at Hospital Discharge,” BMJ Open 9, no. 2 February 2019): e023440. 10.1136/bmjopen-2018-023440 PMC6377507 30782879

