# Supplementary material for: How Did Women From South Asian Backgrounds and People Seeking Asylum and Refugees Look After Their Health and the Health of Others During the COVID‐19 Pandemic? A Community Research Approach
Source: Health Expect. 2026 Jun 21;29(3):e70717. doi: 10.1111/hex.70717 (PMC13283351; doi:10.1111/hex.70717)
Supplement: Supplementary file 1 — Supporting File [file HEX-29-e70717-s001.doc]

**Interview topic guide**

1. Could you tell me a little bit about your general health and well-being?

(Prompts: Current health conditions/ status/ medications)

1. Could you tell me a little bit about how you generally used healthcare services before the COVID-19 pandemic?

(Prompts: Types of services accessed previously/ frequency of access)

1. Could you tell me a little bit about how you usually looked after your general health and well-being before the COVID-19 pandemic?

(Prompts: For example - exercise, healthy diet, not smoking, social support, sleep, keeping hydrated etc.)

1. Could you tell me about when you first heard about the COVID-19 pandemic?

(Prompts: Information sources and trust)

1. Following on from when you first heard about COVID-19, can you tell about any significant time points that were important to you that made you think, reflect or change your behaviour?

(Prompts: Types of information at different timepoints e.g. news updates from the Prime Minister, more general news, Twitter, family, friends, religious settings, community groups, WhatsApp, healthcare workers, Facebook etc; Types of behaviours e.g., social distancing, self-isolation, ability to work, hygiene practices, social engagement)

1. To what extent do you feel that you are able to clearly understand the current advice around COVID-19 and what that means for you?
2. If you had (or have had) symptoms that you thought could be COVID-19, would you know if/when to go to the healthcare service?

(Prompts: Confidence, concerns, seeking different services, challenges and supports)

1. Knowing what you know today, do you think that you can contribute to your own and others’ health and well-being any differently than you did prior to the COVID-19 pandemic?

(Prompts: Wellbeing, safety, access to health services, strengths and weaknesses in current healthcare services, supporting loved ones)

1. Do you think that how you look after your health and well-being will change in the near future?

**Thank you for your time and participating in our study.**
